# Fibrosis in canine transmissible venereal tumor after chemotherapy with vincristine

**DOI:** 10.29374/2527-2179.bjvm000123

**Published:** 2023-04-24

**Authors:** Anderson do Prado Duzanski, Haline Ballestero Feo, Luis Mauricio Montoya Flórez, Fernando Carmona Dinau, Bruna Ribeiro Paiva, Cláudia Valéria Seullner Brandão, Noeme Sousa Rocha

**Affiliations:** 1 Veterinarian, MSc., Faculdade de Medicina, Departamento de Patologia, Universidade Estadual Paulista (Unesp), Botucatu, SP, Brazil.; 2 Veterinarian, DSc., Faculdade de Medicina Veterinária e Zootecnia, Departamento Patologia Investigativa e Comparada, Unesp, Botucatu, SP, Brazil.; 3 3 Veterinarian, DSc., Universidad Nacional de Colombia, Bogotá, Colombia; 4 4 Veterinarian, Programa de Pós-Graduação Biotecnologia Animal, Faculdade de Medicina Veterinária e Zootecnia Unesp, Botucatu, SP, Brazil.; 5 5 Veterinarian, DSc., Faculdade de Medicina Veterinária e Zootecnia, Unesp, Botucatu, SP, Brazil

**Keywords:** dog, CTVT, fibrosis, vincristine, idiosyncratic reaction, cão, CTVT, fibrose, vincristine, reação idiossincrática

## Abstract

The canine transmissible venereal tumor is type of transmissible cancer that occurs naturally through allogenic cellular transplants. Commonly diagnosed in the genital area of sexually active dogs, the tumor typically responds well to vincristine sulfate chemotherapy, although there are cases of resistance to the drug correlated with the tumoral phenotype. We describe herein a case of fibrosis in an area affected by the tumor in a dog after vincristine chemotherapeutic treatment that was associated with an idiosyncratic reaction to the drug.

## Resumo

O tumor venéreo transmissível canino é um tipo de câncer transmissível que ocorre naturalmente através do transplante celular alogênico. Comumente diagnosticado na área genital de cães sexualmente ativos, o tumor normalmente responde bem à quimioterapia com sulfato de vincristina, embora existam casos de resistência à droga correlacionados com o fenótipo tumoral. Descrevemos neste relato de caso um cão com fibrose na área acometida pelo tumor após o tratamento quimioterápico com vincristina associado a uma reação idiossincrática à droga.

## Introduction

Canine transmissible venereal tumor (CTVT) is a documented transmissible type of cancer, which occurs naturally through allogenic cellular transplants, and is commonly diagnosed in the genital area of sexually active dogs that have free access to the street. This type of cancer can affect dogs of different age groups, either by implantation of viable tumor cells in mucous membranes during intercourse, biting, licking, scratching or smell a carrier animal (Ganguly et al., 2016).

Regarding the biological behavior, CTVT as a rule exhibits benign character, however, may present in some conditions characteristic of malignancy. Metastatic processes are uncommon (5%) in cases of CTVT. However, there have been reports in the literature of metastasis in regions such as lymph nodes, kidney, spleen, brain, liver, eye, tonsils, pituitary, skin and subcutis, peritoneum, mesenteric lymph nodes and maxillary bone (Ganguly et al., 2016).The definitive diagnosis of this neoplasm is made by cytological examination, usually from fine-needle aspiration. Furthermore, CTVT tumor cells can also be detected in free-catch samples of urine. In both cases, the tumor cells have a round or oval aspect, exhibiting a distinct appearance, containing multiple clear cytoplasmic vacuoles, mainly during the initial stages of tumor regression. Cytological findings such as mitotic figures and evident nucleoli are also common in this round cell tumor (Ganguly et al., 2016).

The prognosis of dogs affected by CVT is good. Since treatment with Vincristine chemotherapy in most cases results in total tumor regression. In some cases, the prognosis may be worse, due to metastatic processes or chemotherapy resistance (Ganguly et al., 2016).

## Case report

Herein we report a case of fibrosis after chemotherapy in a 10-year-old non-castrated male dog of undefined breed that received a diagnosis of plasmocytoid CTVT in a cytological exam. The dog had been removed from the street by his current guardian and was attended at the Veterinary School Hospital, presenting with a history of bleeding and an increase in penile volume. Clinically, the dog was well, and no other significant finding was noted in hematological or physical exams.

**Figure 1:**
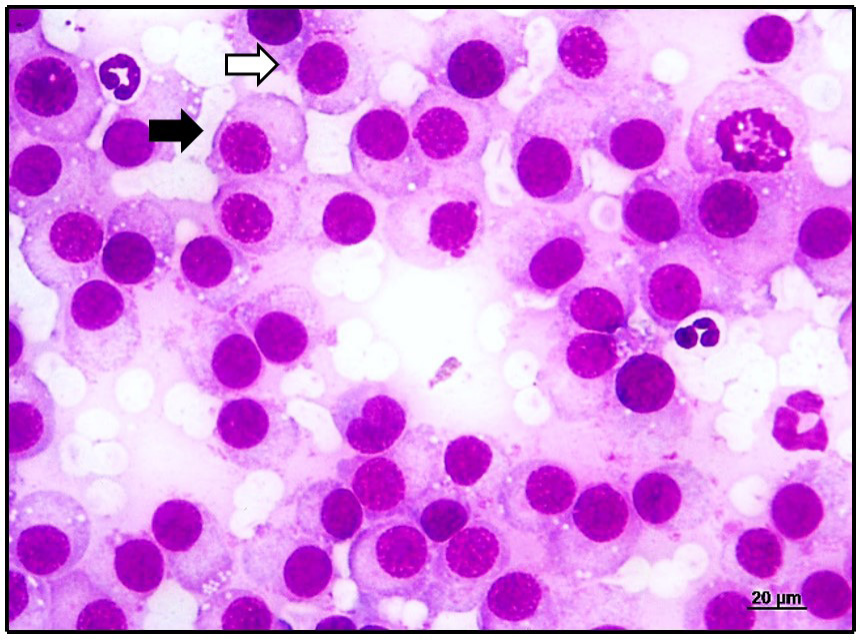
Fine needle aspiration cytology (FNAC) for the diagnosis of CTVT. Plasmacytoid cytological subtypes (black arrow) and lymphocytoid (white arrow). Note the predominance of plasmacytoid oncocytes. Staining by Giemsa, at 63x objective.

**Figure 2:**
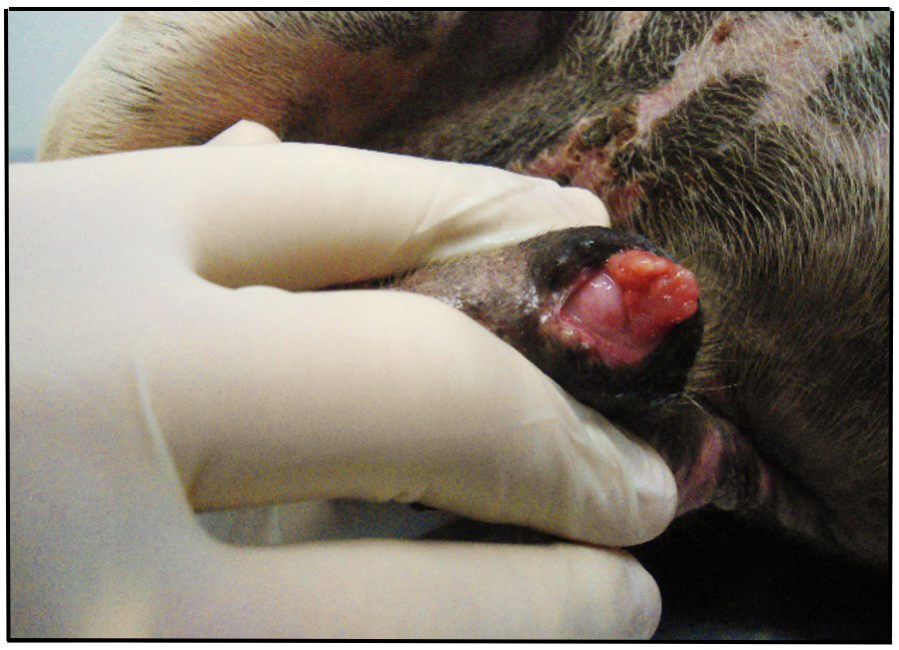
Canine transmissible venereal tumor in a male dog. Multilobular vegetating mass, brittle and of reddish coloration adhering to the skin of the penis before the initiation of chemotherapy. Right ventrolateral view of the animal.

The tumor initially had adhered to the adjacent tissues, preventing exposure of the dog’s penis, and leaving visible only its distal portion in preputial mucosa joined to the glans, which exhibited a friable hyperemic mass of cauliflower aspect (Figure 1). Cytological samples of the exposed zone of the tumor were obtained by fine needle aspiration cytology (FNAC), which revealed high cellularity with a pattern of oval cells, characterizing a plasmocytoid phenotype. The oncocytes presented as individualized, of variable size between 13 and 25 µm, characterized by cytoplasm with distinct delimited contours, slightly basophilic, containing multiple dispersed clear vacuoles. The central or eccentric nuclei, according to the plasmocytoid or lymphocytoid cytological subtype of CTVT, showed aggregated chromatin and distinct nucleoli. Inflammatory infiltrate and mitotic figures were also observed (Figure 2). In addition to cytology, the diagnosis was fully confirmed by means of two other definitive techniques: cytogenetic and histopathological.

**Figure 3:**
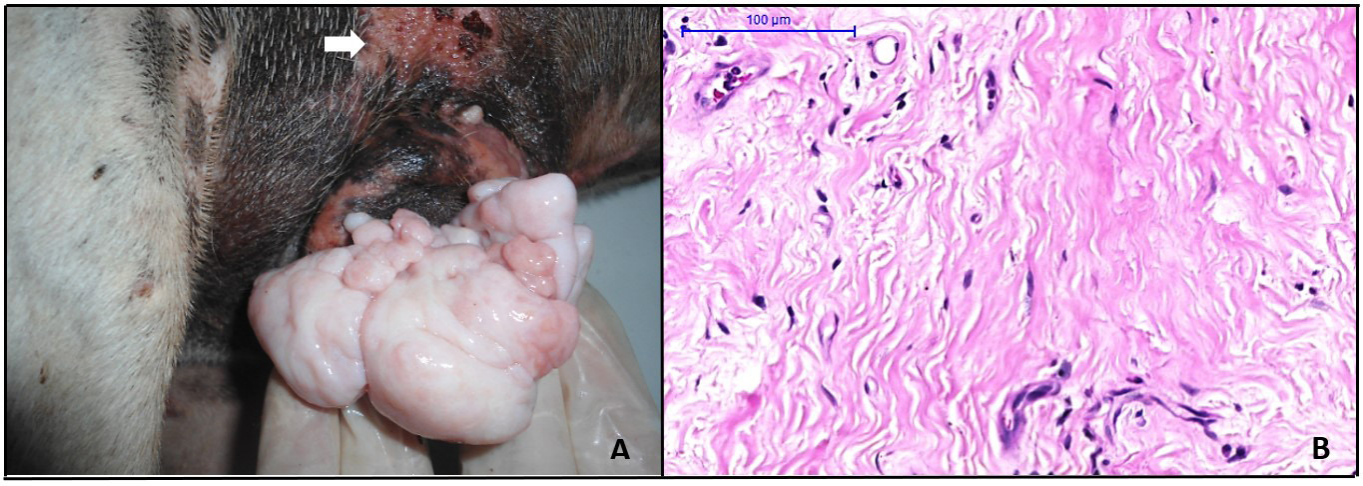
Mass of a dog’s penis after chemotherapeutic treatment. Note the redness on the abdominal skin (white arrow). Right ventrolateral view of the animal (A). Micrograph evidencing fibroblasts dispersed in extracellular matrix and collagenous fibers. Staining by H.E, at 20x objective (B).

**Figure 4:**
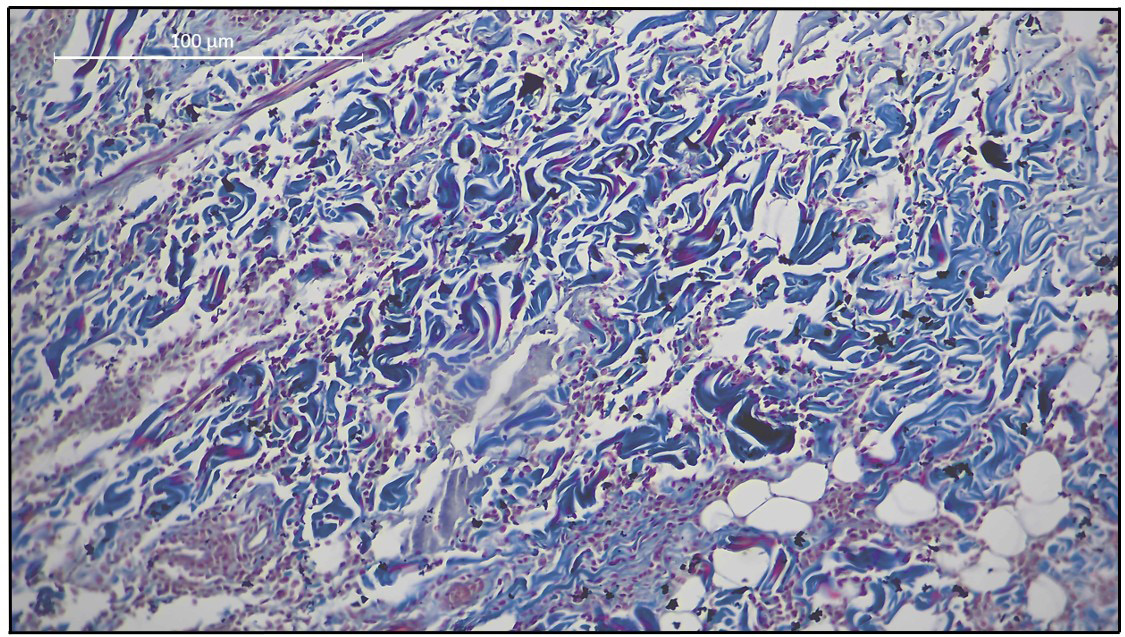
Microscopy showing positive Masson’s trichrome staining for collagen fibers in blue. Representing tissue fibrosis after chemotherapy with Vincristine. Staining by Masson’s trichrome, at 20x objetive.

The treatment instituted was chemotherapy with vincristine sulfate (0.75 mg/m^2^, IV), weekly. Interestingly, after 6 sessions of chemotherapy, upon exposing the penis of the patient to evaluate the clinical evolution of the tumor, we observed the presence of a hardened vegetative multi-lobed mass with a smooth brilliant surface, and of pinkish-white coloration (Figure 3A). The mass was surgically removed and processed by the Veterinary Pathology Service, to make histological slides. In which it was possible to observe a fibrotic process (Figure 3B), confirmed by Masson’s trichrome staining (Figure 4). That when added to the previous histological evaluation was incompatible with the aspect of CTVT.

## Discussion

Studies conducted by a group of oncology researchers from the investigative comparative pathology laboratory at Universidade Estadual Paulista (Unesp), described CTVT as a highly uncommon tumoral type that expresses varied biological behaviors and shows, according to the tumoral phenotype, varied degrees of aggressiveness and significant differences in response to chemotherapy with vincristine (Duzanski et al., 2017).

Vincristine chemotherapy acts on the mitotic apparatus of the cell, and in particular exerts cytotoxic activity by perturbing the formation of cellular microtubules thus inducing the inhibition of cellular replication (Hantrakul et al., 2014). There are reports describing some side effects of vincristine in dogs, citing anorexia, vomiting, diarrhea, depression, hyperesthesia and ataxia (Martins et al., 2014), fur loss and cutaneous ulceration (Andrade, 2002), although hematological toxicity - such as leucopenia, thrombocytopenia and anemia - is the most commonly described side effect (Hantrakul et al., 2014).

Chemotherapeutic agents can eventually cause a direct toxic reaction, whereas the direct toxicity through time can manifest clinically as a picture of fibrosis (Schwaiblmair et al., 2012). In human patients’ cases of inflammation and fibrosis have been reported as a complication of chemotherapeutic treatment (Azambuja et al., 2005; Chan et al., 2011; Schwaiblmair et al., 2012) , above all radiotherapy (Schwaiblmair et al., 2012; Stubblefield, 2011). Fibrosis of organs, such as the lung, are widely described in human medicine, secondary to the use of chemotherapeutic agents such bleomycin, gemcitabine, methotrexate and vincristine sulfate (Chan et al., 2011; Chandler et al., 2019; Do Ki et al., 2010).

In veterinary medicine, a case of pulmonary fibrosis in a Basset Fauve de Bretagne, probably caused by the chronic use of Lomustine, was described (Van Meervenne et al., 2008). However, there has been no mention made of fibrosis associated with vincristine sulfate toxicity in a dog.

In human medicine, the mechanism which vincristine causes pulmonary fibrosis has been related to its ability to promote the differentiation of fibroblasts into myofibroblasts via regulation of the MAPK signal pathway. Therefore, mitogen-activated protein kinase (MAPK) plays a significant role in cell growth and other tissue repair mechanisms associated with fibrotic process (Xu et al., 2022).

**Figure 5:**
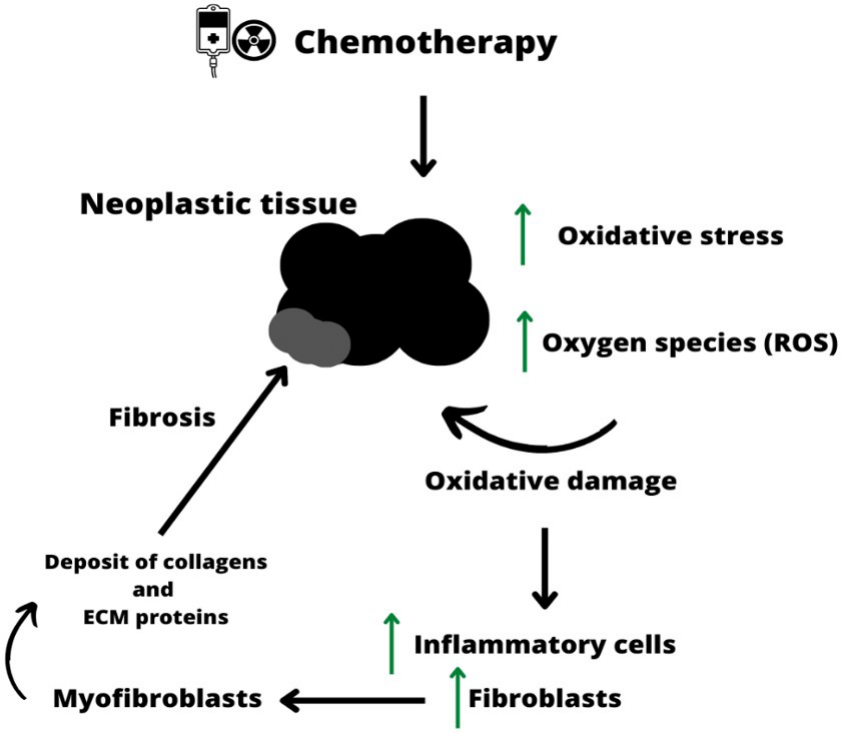
Mechanisms of chemotherapy induced fibrosis: Illustration showing the development of fibrosis during chemotherapy treatment. Evidencing the events that occur during oxidative injury in the neoplastic tissue.

The fibrous tissue forms after fibroblasts, leukocytes and phagocytes that invade the area (MacPhail, 2014). Our current hypothesis is that the fibrosis found in the dog presents a correlation with the chemotherapeutic toxicity of idiosyncratic origin. The cellular lesion caused by oxidative damage during chemotherapy resulted in inflammatory events with exacerbated release of inflammatory chemokines and cytokines. In addition to growth signaling factors, which were capable of communicating and activating the cellular repair biomechanisms, such as fibroblasts and myofibroblasts, causing the process of tissue fibrosis (Borthwick et al., 2012; Mancini & Sonis, 2014) (Figure 5).

We consider that other factors such as the clinical phase of the tumor in the host, as well as the tumoral microenvironment have been strongly mediated by immune factors, including paracrine communication between neoplastic cells and stroma (Ujvari et al., 2016), such as signalization by endothelial growth factor (VEGF) and Mitogen-activated protein kinase (MAPK), which regulates the function and differentiation of fibroblasts (Goel & Mercurio, 2013), plus the possibility of an extra factor, such as a lesion secondary to the tumor, although not identified, may have collaborated in the pathogenesis of the fibrous mass, but they were not the only determinants. We believe that the chemotherapeutic treatment with vincristine sulfate performed a key role in fibrogenesis by an adverse reaction of the dog to the drug. This uncommon and unpredictable event induced by exposure to exogenous agents, such as pharmaceutical products, was already found in a dog (Trepanier, 2004; Voie et al., 2012), and has been described in human patients (Do Ki et al., 2010; Nemery et al., 2001). In the case of the dog’s propensity for medication-dependent toxicity of idiosyncratic genetic predisposition, it is possible that tumor regression during the course of chemotherapy was accompanied by the cytotoxic action of vincristine, or of its metabolites that are reactive to the cells present. The cellular injury activated a cascade of immunological responses, especially those mediated by T cells (Trepanier, 2004), with an exacerbated release of inflammatory chemokines and cytokines, in addition to growth signaling factors (Borthwick et al., 2012), that were capable of communicating and activating cellular repair mechanisms. Although the pathogenesis of idiosyncrasy has not been clarified or elucidated, the syndrome is related to enzymatic defects of genetic origin, occurs independently of dose, and is not directly related to physical, chemical or pharmacological properties of the drugs (Voie et al., 2012).

We also noted that during the chemotherapeutic treatment, the dog presented a cutaneous reaction, characterized by redness in the region of the abdomen and groin, which is considered a common manifestation of the syndrome (Trepanier, 2004; Voie et al., 2012). The chemotherapy was interrupted and the patient was submitted to surgical treatment.

## Conclusion

We believe that the use of vincristine sulfate in this case performed a key role in fibrogenesis by an adverse reaction of the dog to the drug.

However, as the side effect observed in this case was from only one animal, further studies involving the use of vincristine sulfate in dogs must be carried out with the aim of establishing the hypotheses reported here.
